# Extreme *ESR1* Polyclonality, Mutation Dynamics, and Effects on Treatment Outcomes in Patients with ER+ Metastatic Breast Cancer

**DOI:** 10.21203/rs.3.rs-8187308/v1

**Published:** 2026-08-10

**Authors:** Elena Michaels, Tess A. O’Meara, Emily Podany, Marla Lipsyc-Sharf, Annika Putur, Shannon McLaughlin, Teresa T. Ho, Nitin Katakam, Maxwell R. Lloyd, Arielle J. Medford, Charles S. Dai, Laura Spring, Sahar Shahamatdar, Joyce A. O’Shaughnessy, Aditya Bardia, Cynthia X. Ma, Hyo Han, Seth A. Wander

**Affiliations:** 1 Department of Medical Oncology, Mass General Brigham Cancer Institute, Boston, MA, USA.; 2 Harvard Medical School, Boston, MA, USA.; 3 Department of Medical Oncology, Dana-Farber Cancer Institute, Boston, MA, USA.; 4 Broad Institute, Cambridge, MA, USA.; 5 Department of Medical Oncology, Washington University in St Louis School of Medicine, St Louis, MO, USA; 6 Department of Medical Oncology, UCLA Health Jonsson Comprehensive Cancer Center, Los Angeles, CA, USA.; 7 Department of Medical Oncology. H. Lee Moffitt Cancer Center and Research Institute, Tampa, FL, USA.; 8 Department of Medical Oncology, Beth Israel Deaconess Medical Center, Boston, MA, USA.; 9 Department of Medical Oncology, Texas Oncology-Baylor Charles A. Sammons Cancer Center, Dallas, TX, USA.

## Abstract

*ESR1* mutations commonly emerge on endocrine therapy (ET) during breast cancer treatment and contribute to therapeutic resistance. However, the impact of increasing degrees of polyclonality is not well understood. We conducted a retrospective, descriptive review of patients with estrogen receptor-positive (ER+) metastatic breast cancer (MBC) and ≥10 concurrent *ESR1* variants defined as *extreme ESR1 polyclonality*, assessed on circulating tumor DNA (ctDNA) between August, 2020 and July, 2025. Patient and tumor characteristics, frequency of *ESR1* variants, co-occurring mutations, and clonal changes in *ESR1* in response to treatment were explored. Nine patients met criteria for extreme *ESR1* polyclonality. Patients received a median of 1 line of ET in the metastatic setting before their initial ctDNA assessment, which identified 0–31 *ESR1* clones (mean, 6). The most frequent *ESR1* clonal mutations were Y537N, Y537S, and D538G. Common co-alterations included *TP53* and *RB1* mutations and *CCND1* amplification. Tumor mutational burden (TMB) at the time extreme *ESR1* polyclonality emerged ranged from 6.7–56.5 mutations per megabase (mut/Mb); all assessable tumors were microsatellite stable (MSS). This represents the first case series to explore extreme *ESR1* polyclonality. Characterizing the mutational dynamics associated with this phenomenon may improve understanding of *ESR1* mutations and related impacts on treatment resistance.

## Introduction

Estrogen receptor-positive (ER+) breast cancer accounts for approximately 75% of all breast cancer cases. While endocrine therapy (ET) - consisting of an aromatase inhibitor (AI) or the selective estrogen receptor degrader (SERD), fulvestrant - given in combination with a cyclin-dependent kinase 4/6 inhibitor (CDK4/6i) remains the foundation of treatment for advanced ER+ breast cancer, resistance inevitably develops. One well-described mechanism of acquired resistance involves mutations in estrogen receptor-alpha (ER; encoded by *ESR1*), often within the ligand-binding domain (LBD). This leads to constitutive estrogen-independent receptor activation, resulting in AI insensitivity and variable resistance to selective estrogen receptor modulators (SERMs) such as tamoxifen and SERDs.^1–7^ *ESR1* mutations are uncommon in treatment-naïve patients but emerge in 20–40% of patients treated with an AI in the metastatic setting.^1,6,7^

Over the past decade, translational research has expanded our understanding of *ESR1* mutations and enabled the development of novel oral SERDs capable of efficiently binding and degrading both wild-type and mutant ER conformations, thereby mitigating endocrine resistance associated with *ESR1* mutations. Trials of elacestrant and imlunestrant, the two currently approved oral SERDs, demonstrated improved progression-free survival (PFS) in *ESR1*-mutant ER+ metastatic breast cancer (MBC) compared with standard ET after prior treatment with ET plus CDK4/6i.^8–11^ More recently, the SERENA-6 study showed that switching to the oral SERD, camizestrant, upon detection of an *ESR1* mutation after 6 months of AI plus CDK4/6i in the metastatic setting prolonged PFS and delayed time to chemotherapy compared to continuing standard treatment.^12^

Despite these advances, little is known about *ESR1* polyclonality, defined as the presence of >1 concurrent *ESR1* mutation, and the potential impact of multiple *ESR1* clones on the efficacy of novel SERDs.^6,13–16^ *ESR1* polyclonality has been described in several studies and occurs across a diverse array of patients; reported frequencies range from 20–70% among patients with ER+ MBC.^6,13–16^ The impact of *ESR1* polyclonality on prognosis and survival is not clear. One recent real-world study of patients with *ESR1*-mutated tumors treated with elacestrant reported that patients with ≥ 4 concurrent *ESR1* alterations, representing only 11% of a 752-patient cohort, had significantly shorter time to treatment discontinuation and shorter time to next therapy compared to those with a single mutation.^16^ Further, comparison of patients with a single *ESR1* mutation to those with 2, 3, or ≥4 *ESR1* alterations, revealed no differences in clinical outcomes between patients with 1 mutation and those with 2 or 3. These findings suggest that greater degrees of *ESR1* polyclonality may be associated with a more limited response to elacestrant.^16^ In contrast, in SERENA-6, 26/157 patients in the camizestrant plus CDK4/6i arm and 32/158 patients in the AI plus CDK4/6i arm had multiple (>1) *ESR1* mutations detected on ctDNA. Subgroup analysis demonstrated similar benefit from camizestrant plus CDK4/6i among patients with single versus multiple *ESR1* mutations, although the average number of concurrent *ESR1* mutations in this subgroup was not reported.^12^

*Extreme ESR1 polyclonality*, characterized by larger numbers of simultaneous *ESR1* variants, has been observed in clinical practice but has not previously been described in the available literature. Although rare, understanding this phenomenon may provide insight into tumor heterogeneity, endocrine resistance, and clonal evolution.^17^ We, therefore, conducted a retrospective, descriptive multicenter case series highlighting 9 patients with ER+ MBC who developed extreme *ESR1* polyclonality to characterize the clinical and genomic features of their disease.

## Results

### Patient Demographics

Across the 5 participating centers, more than 1,000 patients with ER+ MBC and at least 1 *ESR1* alteration detected on ctDNA were evaluated during the study period. Nine patients met criteria for extreme *ESR1* polyclonality. Among the 227 patients at Mass General Brigham Cancer Institute included in the initial analysis, 134 had a single *ESR1* mutation, 47 had 2 concurrent mutations, 28 had 3, 8 had 4, and 10 had ≥5.

In the 9 patients with extreme *ESR1* polyclonality, the median age at initial diagnosis was 61 years (range, 38–71). Four patients were pre-menopausal and 5 were post-menopausal at diagnosis (Table 1). Eight patients had ER+/HER2- disease and 1 had ER+/HER2+ disease. Five patients received adjuvant chemotherapy (2 with docetaxel-cyclophosphamide and 2 with dose-dense doxorubicin-cyclophosphamide followed by paclitaxel). One patient discontinued chemotherapy after a single cycle due to an early recurrence. Six received adjuvant ET for a median of 5 years (range, 3–7) with tamoxifen (11%), an AI (22%), or sequentially with tamoxifen and AI (33%); 1 patient declined adjuvant ET, 1 recurred before initiating ET, and 1 presented with de novo metastatic disease. No patient received an adjuvant CDK4/6i as all patients were diagnosed prior to their approval in this setting.

One patient had de novo metastatic disease, while 8 developed a metastatic recurrence: 1 recurred within 1 year of initial diagnosis, 2 within 1–5 years, 4 within 6–10 years, and 1 recurred >10 years after initial diagnosis (Figure 1). Six initially presented with bone-only metastases, of whom 5 later developed additional sites of disease. Eight patients received a CDK4/6i in the metastatic setting and 7 were treated with fulvestrant. Four received an oral SERD either with elacestrant or imlunestrant.

### ctDNA Platform

As mentioned above, 90% of ctDNA assessments were performed using the Guardant360 assay. Two patients (patients 7 and 8) underwent testing with the Tempus xF assay at select time points. For patient 7, Tempus xF was used only once at initial metastatic recurrence, with all subsequent assessments performed using Guardant360 (Figure 1G, Supplementary Table 1). Patient 8 underwent Tempus xF testing at 2 time points (test 2/6 and test 6/6), while the remaining assessments used Guardant360 (Figure 1H, Supplementary Table 1).

### ESR1 Variants

Patients received a median of 1 line of ET in the metastatic setting before initial ctDNA testing (Figure 1). The number of *ESR1* variants at first ctDNA evaluation ranged from 0–31 (mean, 6). Extreme *ESR1* polyclonality emerged after a median of 6 years of cumulative ET exposure (range, 1–9). The maximum number of concurrent *ESR1* clones detected on a single ctDNA test was 31.

Across all patients, 95 distinct *ESR1* variants were identified on ctDNA; 17 variants were classified as oncogenic or likely oncogenic, 9 were synonymous mutations, and 69 were VUS (Figure 2).^21,22^ One patient had 10 concurrent *ESR1* fusions (Supplementary Table 1). The most commonly occurring clonal mutations were *ESR1* Y537N, Y537S, and D538G (Figure 2).

### Co-Alterations

The most frequent co-alterations included *TP53* (n=23) and *RB1* (n=14) mutations and *CCND1* amplification (n=11) (Figure 3, Supplementary Table 1). Mutations in *ESR1* Y537N, Y537S, and D538G often co-occurred with *FGFR1* and *CCND1* amplifications while *ESR1* E542D (VUS), H356Y (VUS), and 422del were associated with *TP53* and *APC* mutations. *ESR1* F404I, G415E (VUS), and M421T (VUS) variants commonly co-occurred with *KRAS* mutations.

### TMB and Microsatellite Status

TMB was reported for 5 patients at the time that *ESR1* polyclonality emerged, ranging from 6.7–56.5 mutations per megabase (mut/Mb), median TMB 12.44 mut/Mb (Figure 1). Extreme *ESR1* polyclonality did not consistently correlate with high TMB. One patient (Patient 1) with a TMB of 6.7 mut/Mb had 12 concurrent *ESR1* variants while another (Patient 8) with a somatic *BRCA2* mutation and TMB of 56.5 mut/Mb had 21 *ESR1* variants. The latter received olaparib for 5 months followed by pembrolizumab and remains on immunotherapy more than 18 months later. All 8 patients with reportable microsatellite status were microsatellite stable (MSS). One patient lacked sufficient tumor cells for testing but had a TMB of 7.4 mut/Mb on initial ctDNA.

### ESR1 Mutation Dynamics and Treatment Patterns

In this cohort, a reduction in *ESR1* clone number was observed during or after treatment with capecitabine (Patient 2, 5, 6, 8) and in 1 patient with negative germline *BRCA* testing but a somatic *BRCA2* SS2213Y mutation with a variant allele fraction (VAF) of 22.1% (Patient 8) who received olaparib (Figure 1). In 3 of the patients treated with capecitabine, *ESR1* Y537N, Y537S, and E380Q mutations persisted on ctDNA testing at the time of progression despite an overall decline in clone number. Patient 8 demonstrated complete eradication of *ESR1* clones while on treatment with capecitabine and later redeveloped polyclonality that decreased again with olaparib (21 → 9 clones). Persistent variants included *ESR1* D538G, V422del, F404L, H356D (VUS), and M421I (VUS). However, for patient 8 ctDNA testing was performed with Guardant360 before initiation of olaparib and with Tempus xF after completion of olaparib; differences in assay depth and reporting threshold could have contributed to this finding. Clone numbers remained stable or increased following treatment with eribulin (Patient 6), vinorelbine (Patient 1), and the antibody drug conjugate (ADC), datopotamab deruxtecan (Patient 2) (Figure 1).

Among the 4 patients treated with the oral SERD elacestrant, 3 had extreme *ESR1* polyclonality on ctDNA prior to initiating treatment and 2 had post-treatment ctDNA analyses (Figure 1). Patient 1 showed persistence of *ESR1* Y537C and Y537N mutations with emergence of new E380Q and L536R mutations and an IMPGF1 fusion following elacestrant. Patient 8 developed extreme *ESR1* polyclonality with 17 new *ESR1* variants after elacestrant while several existing clones persisted (D538G, Y537S, H356D [VUS], H524L [VUS]). The median duration on elacestrant was 6 months (range, 4–6). One patient received imlunestrant on a clinical trial for 18 months (Patient 5) and developed new *ESR1* polyclonality at the end of treatment with a persistent *ESR1* Y537S mutation and 14 new variants. She later went on to receive elacestrant. However, ctDNA was not reassessed during or after treatment with elacestrant.

The effect of fulvestrant on *ESR1* polyclonality was variable: 3 patients developed increased *ESR1* clone numbers after fulvestrant-based combination therapy (with CDK4/6i or PI3Kα inhibitor), whereas 2 had a decline in clone number after progression on fulvestrant combined with a CDK4/6i or AKT inhibition. Two others (Patients 3 and 4) had extreme *ESR1* polyclonality after receiving combination therapy with fulvestrant but this was their sole ctDNA assessment. The median cumulative duration of fulvestrant treatment was 24 months (range, 5–41).

At data cutoff, 3 patients had died from progressive disease (15 months, 22 months, and 40 months after metastatic recurrence), 1 transferred care, 1 was in hospice, and 4 remained on active treatment (Figure 1).

## Discussion

This case series represents, to our knowledge, the first description of extreme *ESR1* polyclonality in ER+ MBC. We defined this phenomenon as the presence of ≥10 concurrent *ESR1* mutations detected on ctDNA at any point during a patient’s disease course.^17^ While all of the findings from this hypothesis-generating cohort involving a small group of 9 patients with outlier numbers of polyclonal *ESR1* mutations are purely descriptive in nature and should be interpreted with caution, several notable observations emerged that may provide early insight into this rare and complex pattern of endocrine resistance.

Across this cohort, *ESR1* variants identified on initial ctDNA ranged from 0 to 31 (mean, 6). The emergence of extreme *ESR1* polyclonality occurred after a median of 6 years of cumulative ET exposure (range, 1–9), suggesting that it represents a late event in the trajectory of endocrine resistance. In total, 95 distinct *ESR1* variants and 10 *ESR1* fusions were identified. The majority of these variants are currently classified as VUS, with only a minority confirmed as oncogenic (Figure 2).^21,22^ Among the oncogenic variants, *ESR1* Y537S, Y537N, and D538G were the most frequent activating mutations, consistent with prior studies linking these alterations to ligand-independent ER activation and resistance to AIs and SERMs.^1–7^ Further laboratory modeling and clinical validation is required to determine the functional relevance of the VUS observed in this study.

Co-occurring genomic alterations were common, with *TP53* and *RB1* mutations and *CCND1* amplification being the most frequent (Figure 3, Supplementary Table 1). Distinct co-mutational patterns were observed, suggesting that individual *ESR1* variants may interact differently with other oncogenic pathways to shape patterns of endocrine resistance. Notably, *ESR1* Y537S, Y537N, and D538G, well-characterized activating mutations, frequently co-occurred with *FGFR1* and *CCND1* amplifications, alterations known to promote cell-cycle progression and endocrine resistance through activation of the MAPK and PI3K-AKT signaling pathways.^23,24^ In contrast, *ESR1* E542D (VUS), H356Y (VUS), and V422del were commonly associated with *TP53* and *APC* mutations, while *ESR1* F404I, G415E (VUS), and M421T (VUS) co-occurred with *KRAS* mutations. The frequent co-occurrence of resistance-associated alterations such as *FGFR1* and *CCND1* amplification and RAS/RAF pathway mutations likely reflects an upregulation in estrogen-independent proliferation created by selective pressure from long-term ET exposure.^25^ The association between specific VUSs in *ESR1* with certain co-alterations is unclear. One possible explanation is that these *ESR1* clones may alter the transcriptional activity not only of the ER but also of ER-associated transcription factors resulting in the upregulation of genes in alternative signaling pathways that promote tumorigenesis.^26–27^

The median TMB in this cohort was 12.44 mut/Mb, which is higher than prior studies in ER+/HER2- MBC, where median TMB ranged from approximately 2–6 mut/Mb, depending on treatment history.^28–30^ However, TMB elevation was not consistent across all patients in this study, suggesting that while some tumors with extreme *ESR1* polyclonality may harbor globally hypermutated genomes, such as Patient 8 who had 21 concurrent *ESR1* mutations with a TMB of 56.5 mut/Mb and has had a robust and durable response to pembrolizumab for over 18 months, this is not a universal feature. Our findings indicate that extended time on ET may lead to focused clonal expansion within the *ESR1* gene rather than a generalized increase in mutational burden across the genome. This then raises the question: what is driving that clonal expansion? Changes in *ESR1* clone burden were observed with specific therapies in select patients. However, given the small cohort size, heterogeneous sampling intervals, assay differences, and the absence of contemporaneous measures of tumor burden, no causal or associative inferences can be drawn. In several cases, reductions in detectable *ESR1* variants occurred during or shortly after treatment with capecitabine and olaparib, though the extent to which these reductions reflect a true depletion of *ESR1*-mutant subclones versus a nonspecific decrease in overall ctDNA levels cannot be definitively established. Comparable reductions were not observed with other cytotoxic agents, including eribulin, vinorelbine, and datopotamab deruxtecan. Following exposure to the oral SERDs, elacestrant and imlunestrant, persistence of pre-existing *ESR1* clones and the emergence of additional variants upon progression were noted in 3 patients.

Although exploratory, these findings raise several important biological and clinical questions. Why do certain patients develop extreme *ESR1* polyclonality and what impact does this have on treatment response and outcomes? The phenomenon does not appear to be solely attributable to differences in treatment adherence or elevated TMB. Whether specific biologic features predispose tumors to extensive *ESR1* diversification remains unknown. The optimal definition and quantification of *ESR1* polyclonality also warrants further study. Our threshold of ≥10 concurrent *ESR1* variants was arbitrarily defined; future work may help us refine this threshold to better reflect this patient population. Finally, the observation that certain non-ER–directed therapies - such as capecitabine and olaparib - can reduce *ESR1* clonal burden, whereas oral SERDs may fail to attenuate it, warrants further investigation into how treatment mechanisms may potentially shape the *ESR1* mutational landscape.

Our study has several limitations including its retrospective design, small sample size, and lack of matched tissue sequencing or functional validation of identified variants. The definition of extreme *ESR1* polyclonality as ≥10 concurrent ESR1 variants was operational and intended to highlight the existence and potential clinical relevance of an unusually high degree of ESR1 polyclonality, rather than to establish a biologically validated cutoff. Emerging data from Lloyd *et al*. support the clinical relevance of increasing *ESR1* mutational burden, demonstrating that the presence of ≥4 concurrent ESR1 alterations – an exceedingly rare finding – was associated with inferior outcomes on elacestrant compared to patients with 1–3 mutations.^16^ Future studies in larger validation cohorts will be required to systematically evaluate alternative thresholds across the spectrum of *ESR1* polyclonality including 4–10 or more concurrent variants. In addition, ctDNA assays varied over time and were influenced by differences in assay sensitivity and reporting, which could impact mutation counts and classification. Therefore, direct numerical comparison across assays should be interpreted with caution. Any potential treatment effects were observed in the context of heterogeneous clinical histories and were limited by lack of dynamic tumor volume or tumor fraction information and, thus, no definitive conclusions can be drawn.

Despite these limitations, this study provides an important preliminary look into a rare and underrecognized pattern of *ESR1* heterogeneity and offers a conceptual framework for future investigation. Our case series highlights the complexity of *ESR1* evolution under prolonged endocrine pressure and underscores the need for systematic investigation of clonal diversity as a driver of therapeutic resistance.

## Methods

We performed a retrospective case series across 5 academic medical centers: Mass General Brigham Cancer Institute (Boston, MA), Washington University (St. Louis, MO), Moffitt Cancer Center (Tampa, FL), Texas Oncology-Baylor Charles A. Sammons Cancer Center (Dallas, TX), and UCLA Health (Los Angeles, CA). Eligible patients had histologically confirmed ER+ MBC and ≥10 concurrent *ESR1* variants detected on commercially available next-generation sequencing (NGS)-based circulating tumor DNA (ctDNA) assays between August, 2020 and July, 2025. This threshold was selected pragmatically in the absence of a biologically established cutoff and was defined as extreme *ESR1* polyclonality. Patients with ER+, HER2- and ER+, HER2+ disease were included.

Electronic medical records were reviewed to collect clinical and pathologic data, including age at primary and metastatic diagnosis, menopausal status, tumor histology and receptor status, metastatic sites, and prior lines and duration of systemic therapy i.e. chemotherapy and ET. Serial ctDNA was evaluated to assess the number and type of *ESR1* variants, co-occurring mutations and alterations, tumor mutational burden (TMB), microsatellite status, and dynamic changes in *ESR1* clonality in response to treatment.

The majority of ctDNA assessments were performed using the Guardant360 assay (90%). Both Guardant360 and Tempus xF are FDA-approved assays with established analytical validation.^18–20^ The assays differ slightly in analytic sensitivity and reporting thresholds. The reported limits of detection (LOD) are 0.1–0.5% for single nucleotide variants (SNVs) and insertions/deletions (InDels) with Guardant360, and 0.25% (SNVs) and 0.5% (InDels) with Tempus xF. Variant allele fraction (VAF) reporting thresholds are ≥0.04% (SNVs and InDels) for Guardant 360, and ≥0.1% (SNVs) and ≥0.25% (InDels) with Tempus xF. At comparable VAF thresholds, published data suggest broadly similar sensitivity and specificity across both platforms, suggesting relative concordance between the 2 assays.^18–20^

All reported *ESR1* variants were included in the assessment of *ESR1* polyclonality, irrespective of variant class or predicted functional impact. This included activating mutations as well as synonymous variants and variants of undetermined significance (VUS). Activating mutations were defined as variants classified in OncoKB as “oncogenic” or “likely oncogenic.”^21,22^ The specific variant and type was reported and was classified as missense, nonsense, synonymous, frameshift, InDel, SNV, or copy number gain (CNG). Activating mutations were distinguished from non-activating variants in all figures and tables and are highlighted throughout in bold (Figures 1–2, Supplementary Table 1).

## Supplementary Files

This is a list of supplementary files associated with this preprint. Click to download.

- SupplementaryTable1.pdf

## Figures and Tables

**Figure 1.**
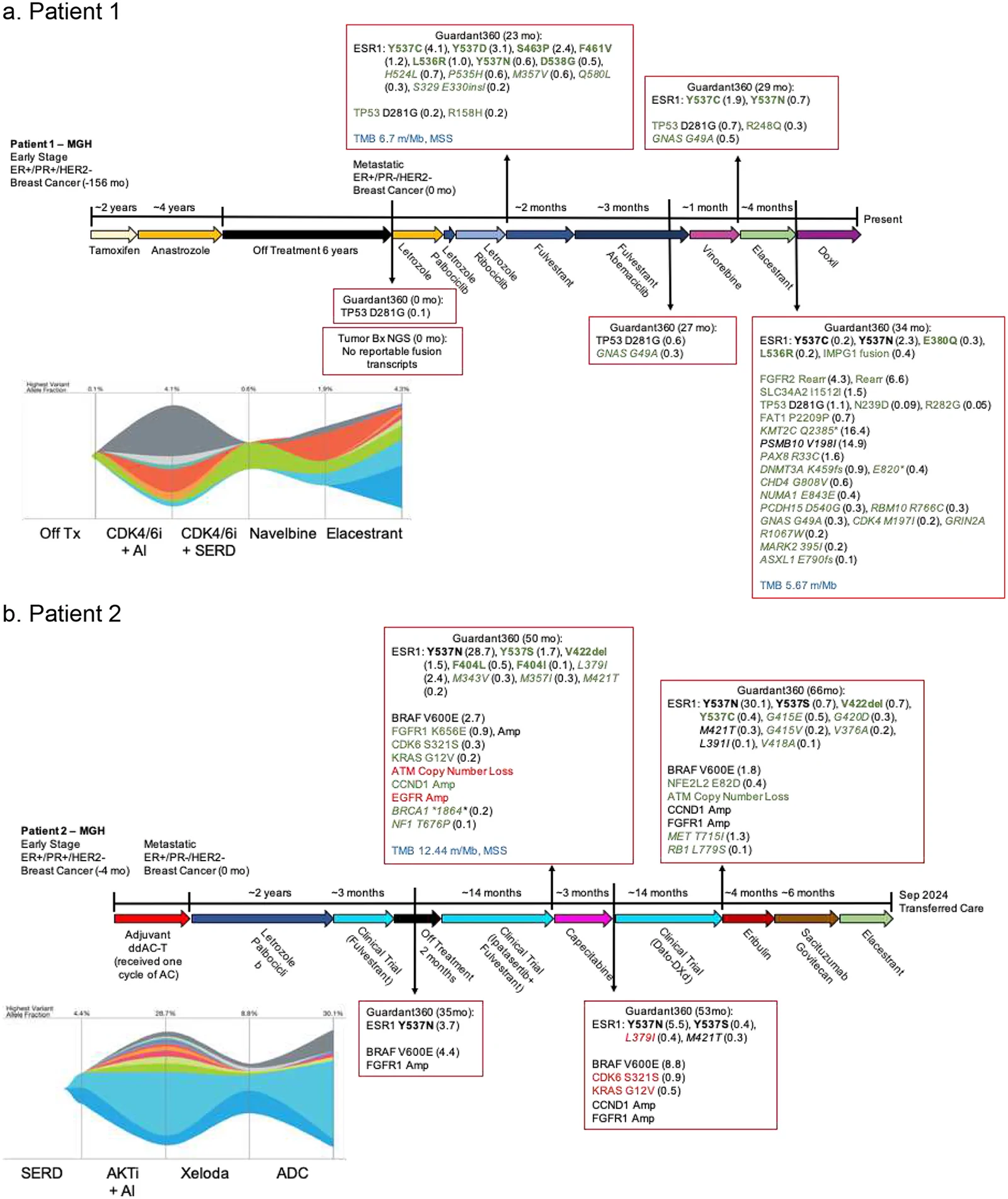

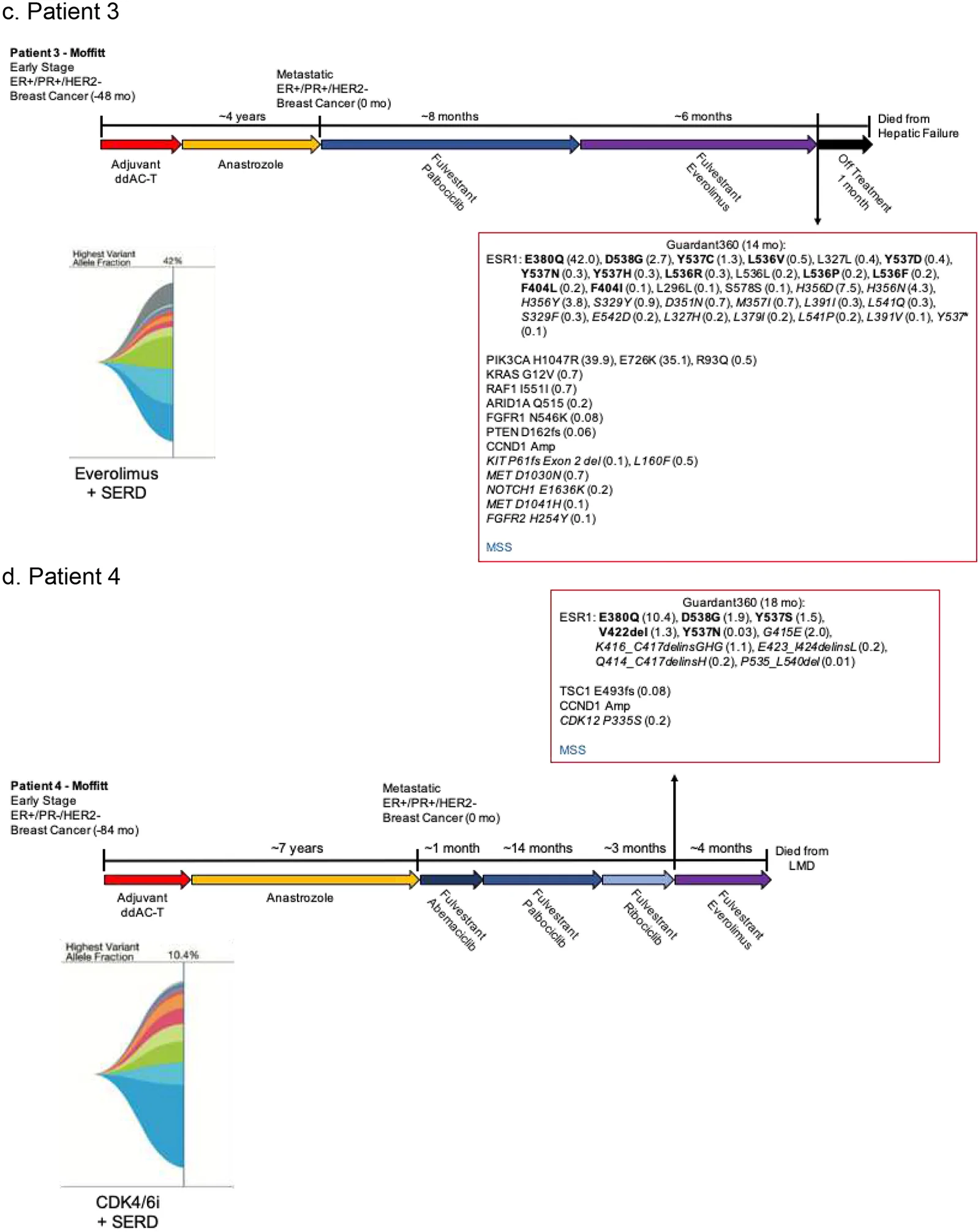

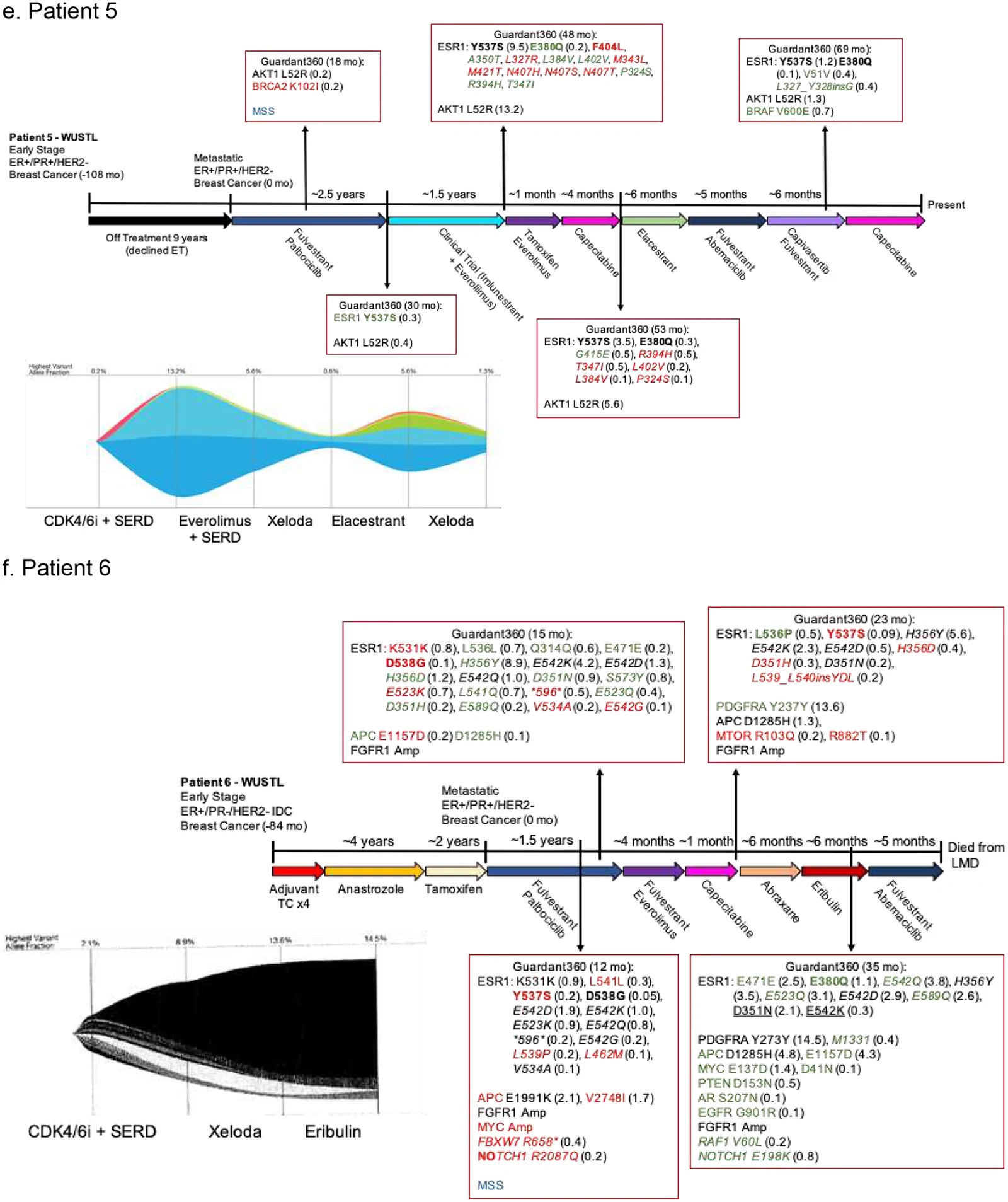

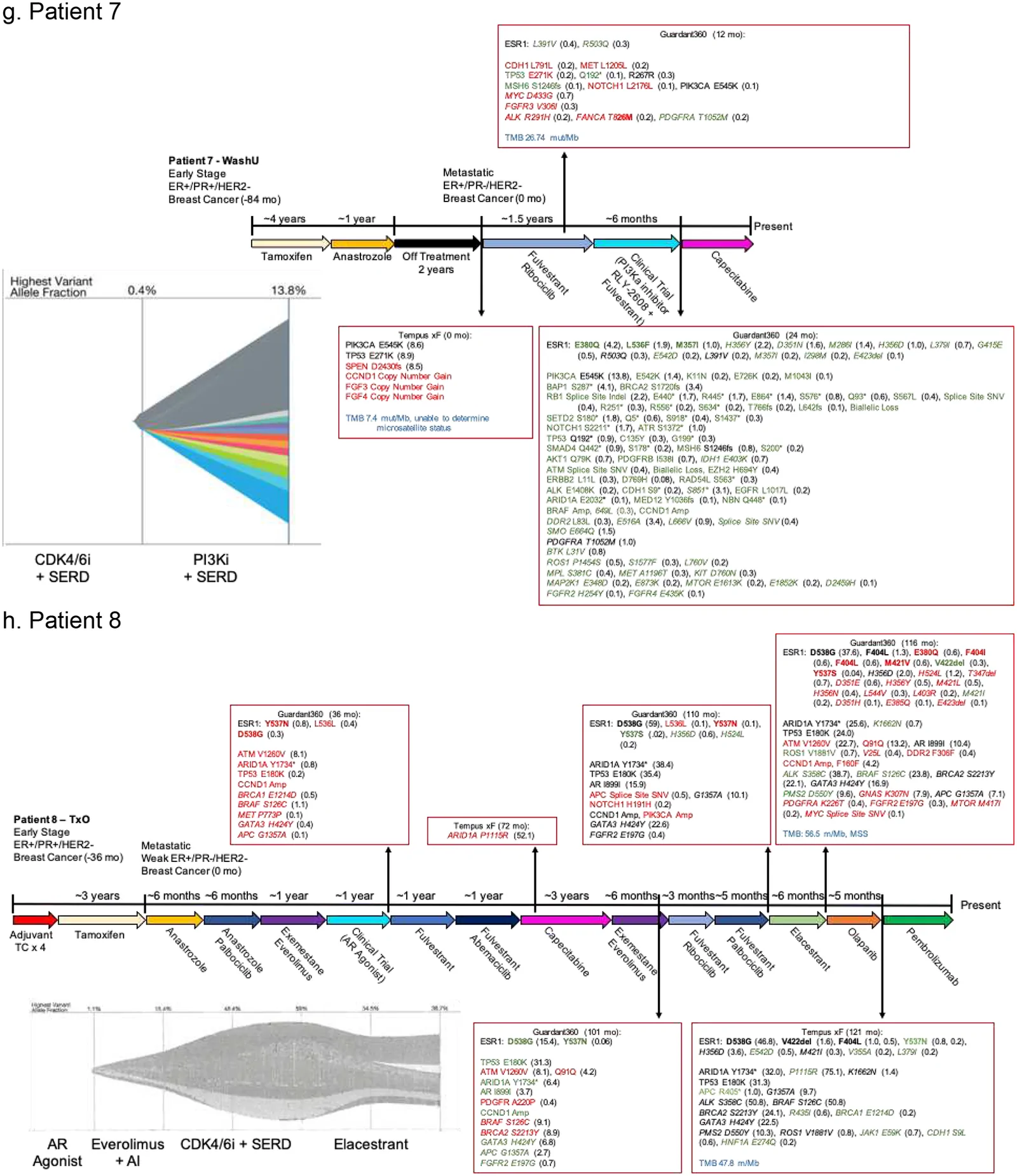

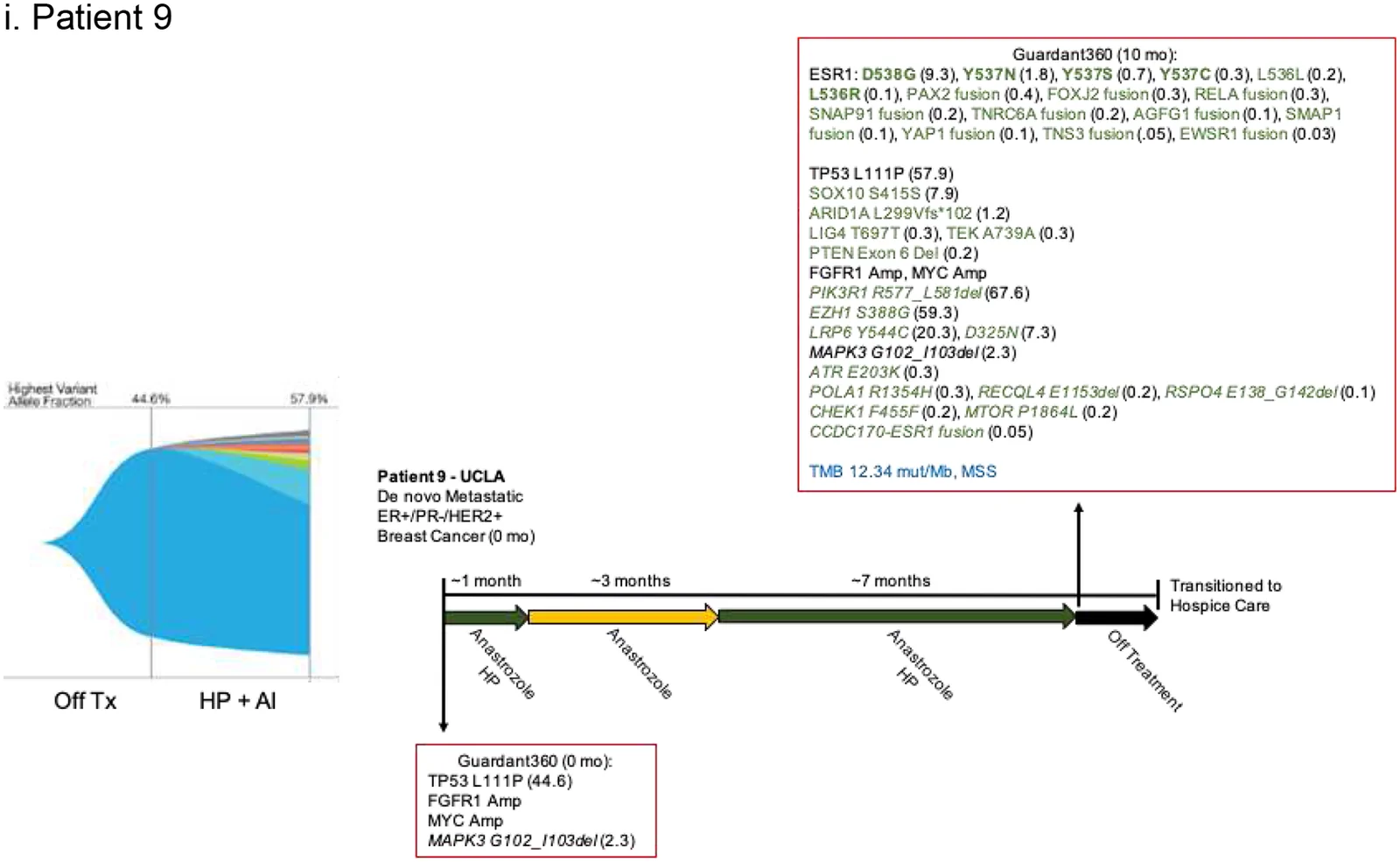
Case vignettes and mutation dynamics Green font = New variant emerged from last ctDNA draw Red font = Variant disappears on next ctDNA draw Blue = Tumor mutational burden and microsatellite status **Bold** = *ESR1* activating mutation *Italics* = Variant of undetermined significance (VUS)

**Figure 2.**
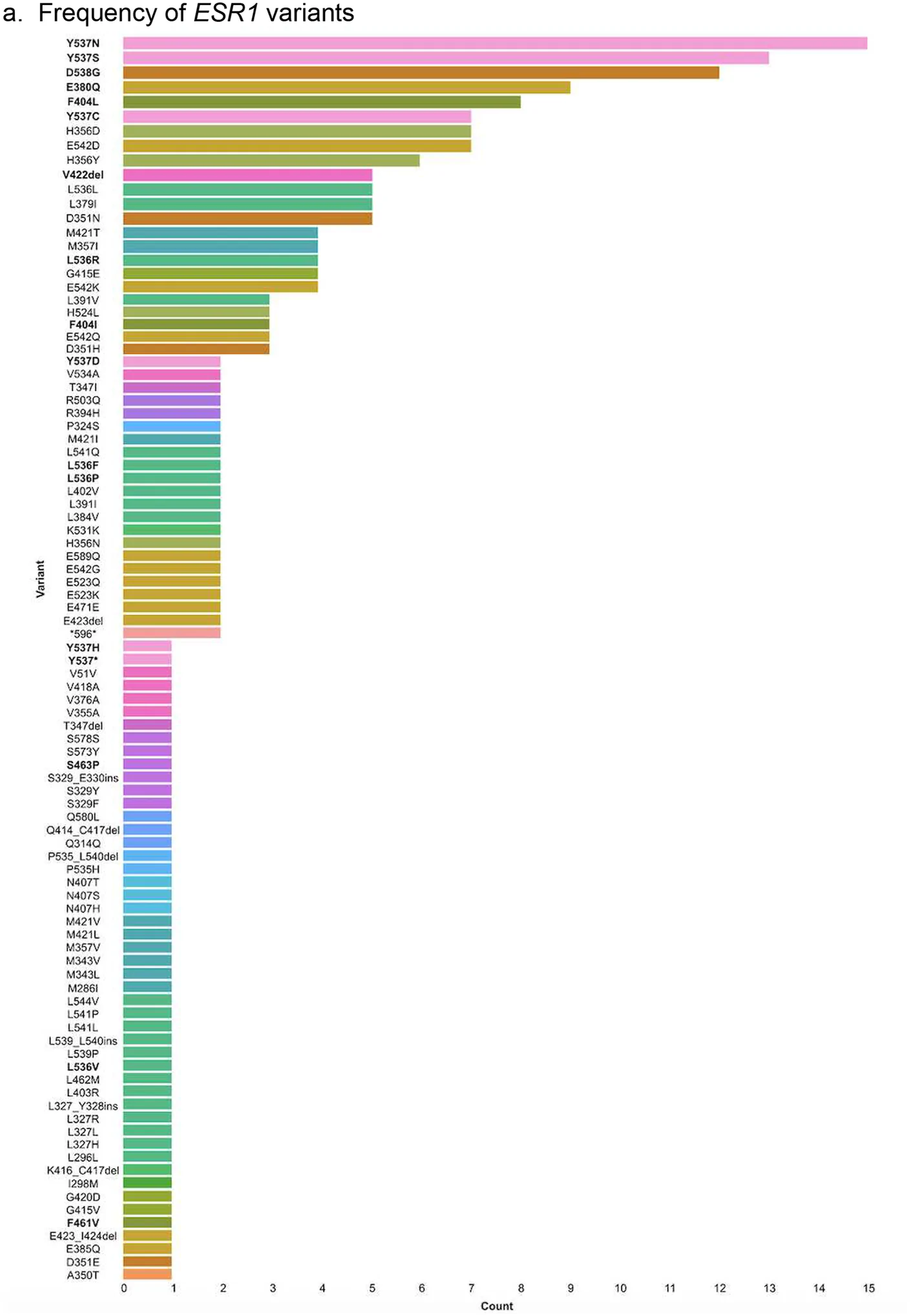

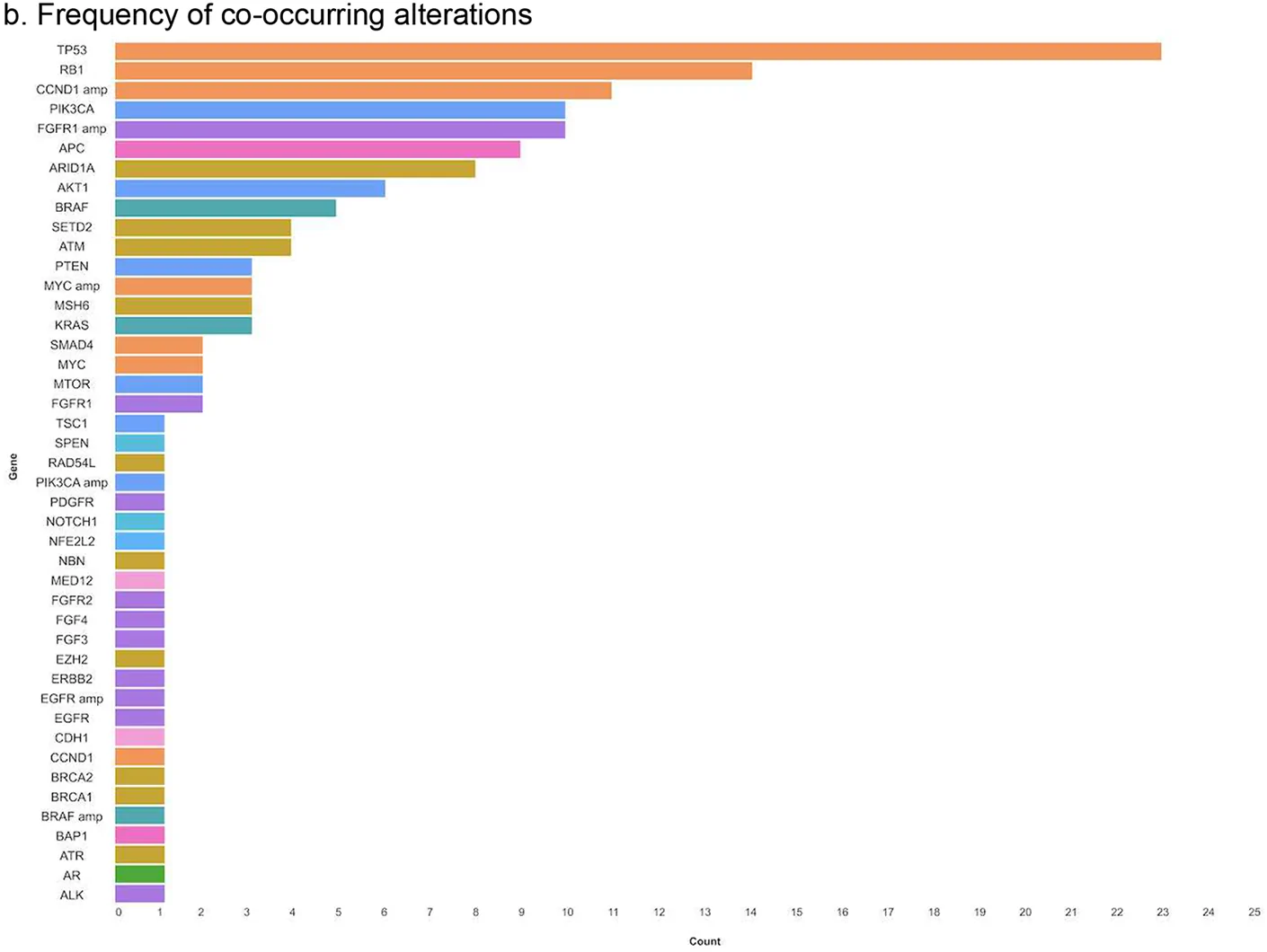
Frequency of *ESR1* variants and associated clones a. Frequency of *ESR1* variants 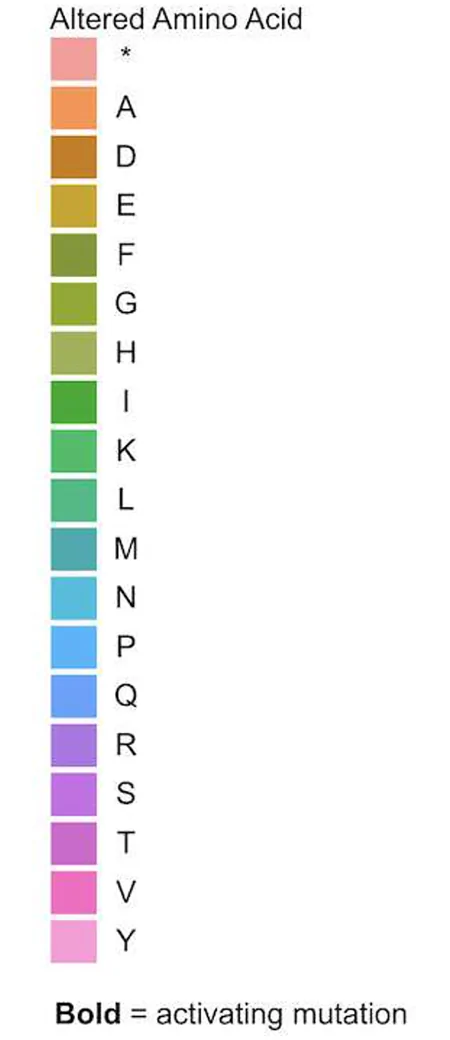 b. Frequency of co-occurring alterations 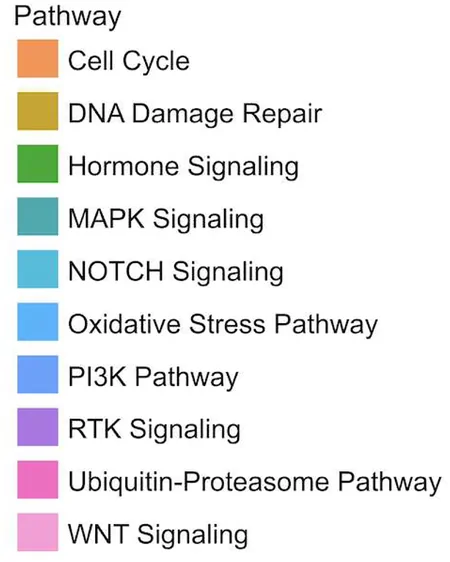

**Figure 3.**
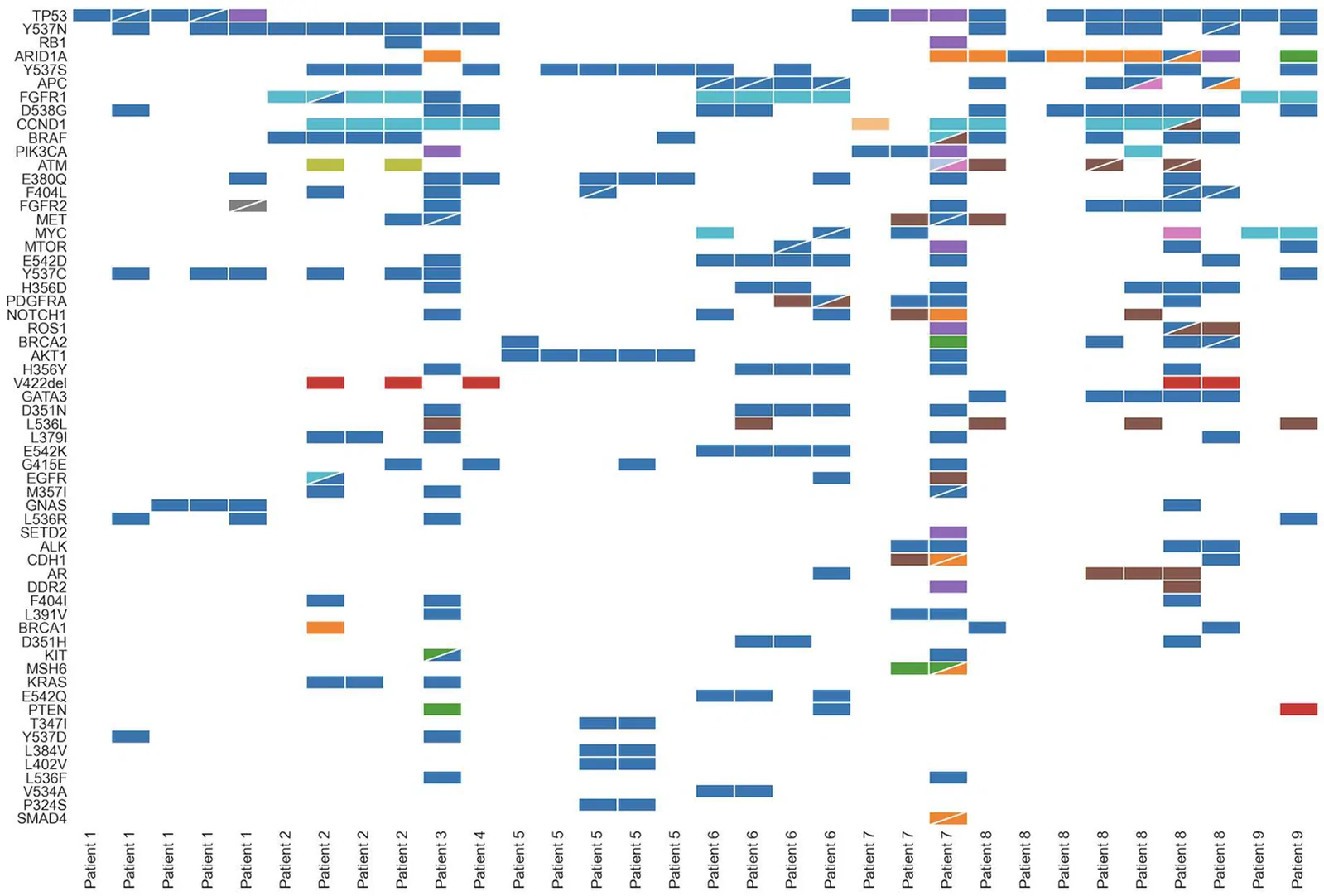
Co-mutation patterns 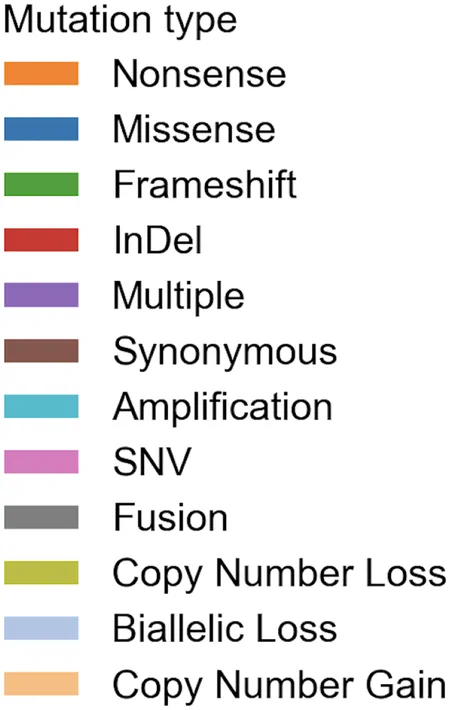

**Table 1.** Patient and tumor characteristics

	Patients (n=9)
Age at metastatic diagnosis (yrs), median (range)	61 (38–71)
Menopause status at primary diagnosis, number (%)	
Premenopausal	4 (44)
Postmenopausal	5 (56)
HER2 by IHC at metastatic diagnosis, number (%)	
0	4 (45)
1+	3 (33)
2+	1 (11)
3+	1 (11)
Adjuvant chemotherapy received, number (%)	5 (56)
Endocrine therapy (ET) received in adjuvant or metastatic setting, number (%)	9 (100)
Years of adjuvant ET, median (range)	5.5 (3–7)
De novo metastatic disease, number (%)	1 (11)
Bone only disease at metastatic diagnosis, number (%)	6 (67)
Lines of therapy received in metastatic setting, median (range)	6 (1–13)
CDK 4/6 inhibitor received in metastatic setting, number (%)	8 (89)
Injectable SERD received in metastatic setting, number (%)	7 (78)
Oral SERD received in metastatic setting, number (%)	4 (44)
Total years of ET prior to emergence of extreme *ESR1* polyclonality, median (range)	6 (1–9)
Tumor mutational burden (TMB), median (range)	12.44 (6.7–56.5 mut/Mb)
Microsatellite status, number (%)	
Microsatellite stable	8 (89)
Microsatellite unstable	0
Not reported	1 (11)

## Data Availability

All data generated or analyzed during this study are included in this published article [and its supplementary information files].
